# Genetic variation and demographic history of Sudan desert sheep reveal two diversified lineages

**DOI:** 10.1186/s12864-023-09231-6

**Published:** 2023-03-16

**Authors:** Bashir Salim, Saeed Alasmari, Nouh Saad Mohamed, Mohamed-Khair A. Ahmed, Ryo Nakao, Olivier Hanotte

**Affiliations:** 1 Department of Parasitology, Faculty of Veterinary Medicine, University of Khartoum, P.O Box 32, Khartoum-North, Sudan; 2 Department of Biology, Faculty of Arts and Sciences, Najran University, 1988 Najran, Kingdom of Saudi Arabia; 3 Molecular Biology Unit, Sirius Training and Research Center, Khartoum, Sudan; 4 Department of Genetics and Animal Breeding, Faculty of Animal Production, University of Khartoum, Khartoum, Sudan; 5 Laboratory of Parasitology, Faculty of Veterinary Medicine, Graduate School of Infectious Diseases, Hokkaido University, Sapporo, Japan; 6 Cells, Organisms and Molecular Genetics, School of Life Sciences, University of Nottingham, Nottingham, UK; 7 LiveGene – CTLGH, International Livestock Research Institute, Addis Ababa, Ethiopia

**Keywords:** mtDNA, Sheep, Haplogroup B and A, Hamary, Kabashi

## Abstract

More than 400 million sheep are raised on the African continent, the majority of which are indigenous and are primarily reared for sustenance. They have effectively adapted to various climatic and production environments, surviving and flourishing. The genetic relationships among these sheep populations remain understudied. Herein, we sequenced the entire mitochondrial DNA control region of 120 animals from Hamary and Kabashi and their crossbreed (Hamary x Kabashi) of Sudan desert sheep (SDS) to understand their maternal-inherited genetic variation and demographic history profiles and relate those to the history of sheep pastoralism on the African continent. The results show a diversified and predominant *D*- loop haplogroup B (n = 102, 85%), with all other sequences belonging to haplogroup A. Most of the maternal genetic variation was partitioned between haplogroup (76.3%) while within haplogroup accounted for 23.7% of the variation. However, little genetic differentiation was observed among the two breeds and their crosses, with our results supporting a Hamari maternal origin for the crossbreed. Bayesian coalescent-based analysis reveals distinct demographic history between the two haplogroups, two breeds and their crosses. Comparison of the two haplogroup showed that haplogroup B experienced an earlier expansion than haplogroup A. Unlike the breed-based comparison, the expansion of the two breeds started roughly at the same time, around 6500 years ago, with Kabashi having a slightly greater effective population size. The maternal ancestors of SDS may have diverged before their introduction to the African continent. This study provides novel insights into the early history of these two main breeds of Sudan desert sheep and their crosses.

**Supplementary Information:**

The online version contains supplementary material available at 10.1186/s12864-023-09231-6.

## Introduction

Countless rural households and pastoralists across the African continent rely on indigenous sheep breeds for their livelihood. These breeds are thought to contain unique genetic variations that allow them to tolerate adverse environmental circumstances. They do well in ecologically marginal areas like the mountainous, desert, and semi-arid areas where other domestic animals might not economically survive [1]. Both genetic data from modern and archaeological specimens highlight our understanding of animal domestication. It is well documented that the main ancestor of domestic sheep (*Ovis aries*) belongs to a species found in the Fertile Crescent, the Asiatic mouflon *Ovis orientalis* [2]. Historical genetic profiles of sheep have been investigated by analysing maternally inherited mitochondrial DNA (mtDNA) in modern sheep breeds. Hitherto, there are at least five genetically different lineages. Sheep belonging to haplogroups A and B are present in many parts of the world, and haplogroups C, D, and E, have a much more restricted geographical range [3, 4]. These different lineages might represent spatially and temporally discrete “domestication events” in which diverse populations of animals were brought under domestication independently of one another [5]. According to a study by Tarekegn and his colleagues [6], sheep and goats first entered Egypt through the Sinai Peninsula, the Mediterranean and the Red Sea coast before spreading through the Nile Basin southward into Sudan and Ethiopia. Until now, no mutation rate of the sheep control region has been documented. However, complete mtDNA dating suggests 30 million years ago for the divergence between the bovine and ovine lineages [7]. Also, a recent study [8] showed that the male-specific region of the Y chromosome has 0.93 × 10–10 mutations per generation per site, which is roughly fifty times the one reported for the full mtDNA.

Sudan desert sheep belong to the thin-tailed hair sheep group and subgroup of African long-legged sheep. They are found strictly within the semi-arid climatic zone of Sudan, North of the 10 degree north latitude, extending eastwards into Eritrea and westwards into Chad (DAGRIS). The Sudan Desert sheep probably descended from ancient Egyptian stock [9]. These sheep originated in western Asia and entered Africa through the Isthmus of Suez. Until the third Millennium BC, the only type of sheep on the African continent was the hairy thin-tailed sheep. Domestic sheep had reached Egypt and other parts of North Africa by 5000 BC. The today observed coat colours of tribal breeds might have been already present in the ancestral population with selection toward colours preferred by particular groups or tribes leading to the near fixation of coat colour in some populations. For instance, Hamari breeds in south-western Kordofan and south-eastern Darfur are predominately brown and dark brown, whereas the Kababesh sheep (Kabashi) of northern Kordofan and northern Darfur are multi-coloured [10]. According to the Veterinary Legislation Identification Mission Report, the sheep population of Sudan was estimated to be approximately 39.2 million in 2016 (REF). In 2017 40.752.000 heads of sheep have been reported [11] (www.ceicdata.com/en/sudan). Whereas a report published in 2018 indicated that Sudan in 2009 had about 51.5 million sheep with a total meat production of 313,000 tons [11].

This study investigated the maternal genetic variations and demographic histories of three indigenous and important Sudan desert sheep breeds by analysing the mitochondrial DNA (mtDNA) control region. To better understand sheep pastoralism in North-East Africa, its origins and evolution, we particularly sought to assess the maternal genetic diversity, and its variation within and among the Hamary, Kabashi and their crossbreed (Hamary x Kabashi) breeds.

## Materials and methods

### Sampling and DNA extraction

A total of 120 blood samples from Sudan desert sheep breeds (Hamary, N = 72; Kabashi, N = 25; and crossbred, N = 23) from North Kordofan State was collected. These animals were owned by nomads who had no records of their pedigree or book registration. The owner, however, knew off-hand the breed origin. To avoid sampling sibling or related animals, we sampled different herds. Informed consent has been obtained from all the owners, and all efforts were made to avoid sampling closely related individuals. The sampling protocol was approved by the Faculty of Veterinary Medicine, University of Khartoum, according to their guidelines for sampling domestic animals in Sudan and in accordance with ARRIVE guidelines (https://arriveguidelines.org). Genomic DNA was extracted using DNeasy® Blood and Tissue Kit (Qiagen, Germany), following the manufacturer’s instructions.

### PCR amplification and sequencing

Complete mtDNA D-loop region (1180 bp) was amplified using forward primer CsumF was 5’GGCTGGGACCAAACCTAT − 3’, and the reverse primer CsumR was 5’-GAACAACCAACCTCCCTAAG − 3’ as described by [12]. PCR reactions were performed in a 25 µl-reaction mixture containing 12.5 µl of 2 × Gflex PCR Buffer (Mg2+, dNTP plus) (TaKaRa.

Bio Inc., Shiga, Japan), 0.5 µl of Tks Gflex DNA polymerase (1.25 units/µl) (TaKaRa Bio Inc.), 200 nM of each primer, and 1.0 µl of template DNA. The thermal reaction conditions consisted of an initial denaturation step at 95 °C (3 min), followed by 35 cycles of 95 °C for 1 min, 56 °C for 30 s, and 68 °C for 90 s, and a final extension step at 68 °C for 5 min. PCR products were purified by using a NucleoSpin Gel and PCR Clean-Up Kit (Takara Bio Inc.) and sequenced directly by the two PCR primers using the BigDye Terminator version 3.1 Cycle Sequencing Kit (Applied Biosystems, Foster City, CA, USA). The sequencing was analyzed on an ABI Prism 3130 x genetic analyzer (Applied Biosystems) according to the manufacturer’s instructions.

### Sequence data analysis

Prior to analysis, all the chromatograms were visually inspected, and sequence fragments were manually edited using ATGC software version 9.1 (GENETYX Corporation, Tokyo, Japan), to correct base-calling errors. Multiple sequences alignments were performed using MUSCLE algorisms implemented in MEGA 7 [13], reference sequence to each haplogroup was utilized ([7] haplogroup A and B; AF039578 and AF039577 [14] for haplogroups C, D and E; HM236178, HM236180 and HM236182). These were subsequently joined to reconstruct a 1180 bp fragment spanning the entire ovine mtDNA D-loop. The haplotypes were determined with DnaSP v5 [15]. The data processing was performed based on haplogroups and breeds. The level of genetic diversity was determined by the number of haplotypes, haplotype diversity, nucleotide diversity, and mean numbers of nucleotide differences between haplotypes. This was computed for haplogroup and breed datasets using Arlequin 3.5 [16]. To gain insight into the genetic relationships between the haplotypes and determine the number of distinct mtDNA D-loop haplogroups present in the dataset, a median-joining (MJ) haplotype network [17] was created using PopArt software 1.7 (https://popart.maths.otago.ac.nz). All the mutations and character states were weighted equally.

The Analysis of Molecular Variance (AMOVA) was performed in Arlequin v3.5 with 1,000 permutations to partition the genetic variation among populations and sub-populations. Phi (*φ*) statistics representing haplotype correlations at various hierarchical levels (*φ*CT, *φ*SC, *φ*ST) were calculated. The significance levels of the variance components associated with the different hierarchical clusters were evaluated with 1000 nonparametric coalescent simulations in Arlequin v3.5 [16]. The sequences obtained and analysed in the study were submitted to the DNA Data Bank of Japan (http://www.ddbj.nig.ac.jp) under accession numbers LC456425 – LC456544.

### Mismatch distribution, tests of neutrality, and bayesian inferences

Each population’s historical dynamics and demographic profiles were inferred from mismatch distribution patterns [17]. The chi-square test of goodness of fit and Harpending’s raggedness index “*r*” [18] statistics were used to evaluate the significance of the deviations of the observed sum of squares differences (*SSD*) from the simulated model of expansion (demographic or spatial) following 1,000 coalescent simulations. Fu’s *Fs* [19] and Tajima’s *D* [20] statistics were also calculated using the infinite sites model in Arlequin v3.5 to supplement the mismatch distributions. To further explore the evolutionary relationships between breeds, the unrooted neighbour-joining (NJ) phylogenetic was reconstructed using MEGA 7.

The demographic dynamics and history of the two breeds and their crosses were further investigated by generating Bayesian Skyline Plots (BSP) [21] using the piecewise constant function implemented in BEAST 2.0 [22] following [23]. In brief, the HKY + G + 1 nucleotide substitution model was used for the analysis, and each Markov Chain Monte Carlo simulation (MCMC) run was performed for 2000 million generations that were sampled every 2,000 generations. The initial two million generations served as burn-in. Convergence of the posterior estimates of the *N*_e_ to the likelihood stationary distribution was evaluated with TRACER v1.6 (http://tree.bio.ed.ac.uk/software/treestat/). Since there is no available mutation rate for sheep D-loop, we calibrated the BSPs using the molecular rate of evolution (µ) of cattle mtDNA D-loop of 6.94 × 10 − 7 substitutions/site/year [s/s/y; 95% highest posterior density interval (HPD) 4.52 × 10 − 7– 9.35 × 10-7s/s/y] [24]. The final BSP plot was generated using outputs from TRACER v1.5 and displayed using MICROSOFT EXCEL (Microsoft Corporation).

## Results

### Sequence variability and diversity analysis of the two lineages and the breeds

One hundred and twenty sequences, spanning the 1180 bp of the ovine mtDNA D-loop, were generated (Hamary, Kabashi, and crossbreed (Hamary x Kabashi). Following their alignment against the reference sheep sequence of haplogroup lineages, two haplogroups, A and B, were identified. The complete mtDNA control region sequences were obtained, spanning the *Ovis aries* reference for 120 sequences. These sequences show 175 polymorphic sites, a transversion to transitions rate of 133:4 and two indels for haplogroup B, and a transversion to transitions rate of 35:2 and two indels for haplogroup A. The total haplotype and nucleotide diversities were 0.993 and 0.08, respectively. The analysis of mtDNA lineages A and B revealed a high level of nucleotide diversity differences between haplogroups (K = 44.748) and a low level of nucleotide substitution per site between haplogroups (0.03792). The predominant haplogroup B included 102 individuals and 79 haplotypes, whereas haplogroup A consisted of 18 individuals and 17 haplotypes (Table 1). The number of haplotypes detected in each Sudan desert sheep population was 64 (88.88%), 24 (96%), and 17 (74%) for Hamary, Kabashi, and the Crossbreed, respectively High haplotype and low nucleotide diversity were observed in the three breeds (Table 2), supporting high levels of maternal genetic diversity for the three Sudan desert sheep populations examined.

**Table 1:** Complete D-loop region of the mtDNA diversity between two lineages and the three of Sudan desert sheep

Population	N	S	H	Hd (SD)	π (SD)	K	*D*	*Fs*	SSD (*P-value*)	‘*r*’ (*P-value*)	K^b^*	D^C^
**Haplogroup A**	18	36	17	0.994 (0.021)	0.007 (0.004)	11.266 (5.155)	-0.829 ( 0.221)	-8.556 (0.001)	0.019 (0.060)	0.031 (0.300)	**44.748**	**0.03792**
**Haplogroup B**	102	139	79	0.992 (0.003)	0.009 (0.005)	8.569 4.155	-1.917 (0.005)	-24.334 (0.000)	0.0007 (0.570)	0.004 (680)		
**Total**	120	175	96	0.993	0.008							

* N*, sample size; S, number of polymorphic sites; H, number of haplotypes; Hd, haplotype diversity; π, nucleotide diversity; K, mean number of nucleotide differences; SD, standard deviation. *D*, Tajima’s D; *Fs*, Fu’s Fs; *SSD*, Sum of square deviation, ‘*r*’, Harpending’s raggedness index, Kb*; Average number of nucleotide differences between Haplogroups, DC; Average number of nucleotide substitution per site between Haplogroups

**Table 2:** Complete D-loop region of the mtDNA diversity between the two of Sudan desert sheep and crossbreed

Breed	N	Hap-A	Hap-B	S	H	Hd (SD)	π (SD)	K (SD)	*D*	*Fs*	SSD (*P-value*)	‘r’ (*P-value*)
**Hamary**	72	12	60	151	64	0.996 (0.003)	0.018 (0.009)	21.415 (9.555)	-1.088 (0.127)	-24.064 (0.000)	0.012 (0.100)	0.004 (0.770)
**Kabashi**	25	5	20	100	24	0.997 (0.009)	0.019 (0.009)	22.160 (10.103)	-0.73 (0.241)	-7.561 (0.007)	0.018 (0.310)	0.015 (0.270)
**Crossbreed**	23	1	22	78	17	0.968 (0.006)	0.011 (0.006)	13.130 (6.135)	-1.569 (0.041)	-3.298 (0.099)	0.008 (0.390)	0.020 (0.440)
**Total**	**120**	**18**	**102**									

*N*, sample size; S, number of polymorphic sites; H, number of haplotypes; Hd, haplotype diversity; π, nucleotide diversity; K, average number of nucleotide differences; SD, standard deviation. *D*, Tajima’s D; *Fs*, Fu’s Fs; *SSD*, Sum of square deviation, ‘r’, Harpending’s raggedness index

### Population phylogenetic analysis and partitioning of genetic variation

**Fig. 1:**
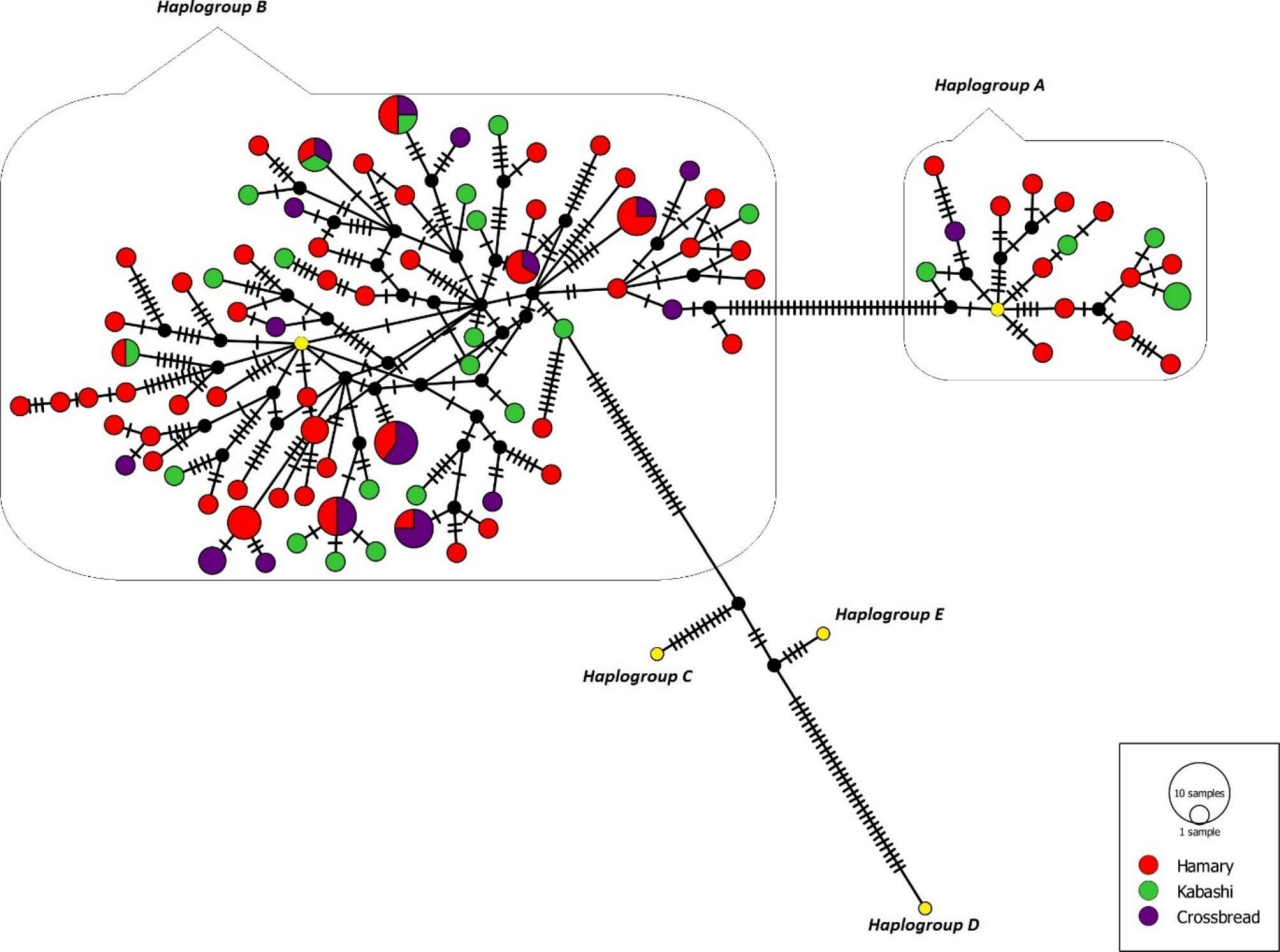
Median joining network showing the relationships among 96 Sudan desert sheep haplotypes. 17 belongs to Haplogroup A, 79 belongs to Haplogroup B. Reference sequences are represented in yellow colour, red, green and purple colours denoted for Hamari, Kabashi and Crossbreed. None of the them belongs to Haplogoups either C, D E

We constructed a median-joining haplotype network to understand the phylogenetic relationships of Sudan desert sheep based on the complete mtDNA D-loop sequences of 120 individuals. Using the reference sequences for the five sheep haplogroups (A, B, C, D and E) (Fig. 1), the sequences were clustered into two main haplogroups, A and B with a total of 96 distinct haplotypes.

Haplogroup B was the predominant haplogroup. A total of 96 haplotypes were identified, of which 69 haplotypes were singletons and eight haplotypes were shared among the breeds within haplogroup B, whereas in haplogroup A, 16 haplotypes were singletons, and one shared haplotype was within Kabashi breed (Table 1). The commonest haplotype included five individuals (3 Crossbreed and 2 Hamary). The next most common haplotype was composed of four individuals each (Hamary, Kabashi, and Cossbreed) (Fig. 1). The number of shared mutations between haplogroup is 21, and the number of net nucleotide substitutions per site between haplogroup was Da = 0.02948. As is shown in (Figure S1), the NJ phylogenetic tree revealed that 79 haplotypes of SDS sequences clustered into haplogroup B, and the remaining 17 into haplogroup A. The haplotype network analysis showed a star-like structure for haplogroup B, suggesting population expansion.

We also examined the genetic distance between the two haplogroups and among breeds, measured in nucleotide substitutions per site, by dividing the three breeds of Sudan desert sheep populations into two groups using the neighbour-joining phylogenetic tree constructed from the 120 sequences of the mtDNA control region (Figure S1). The AMOVA analysis at the breed level resulted in little genetic differentiation among the three breeds (Table S1). However, AMOVA revealed a clear genetic distinction between the two haplogroups with 76.3% of the variation between haplogroup and 23.7% within haplogroup (Table 3). These results support a high maternal genetic differentiation between the haplogroups for Sudan desert sheep. The comparison also revealed 15 polymorphic sites in haplogroup A, monomorphic in haplogroup B and 116 polymorphic sites in haplogroup B, but monomorphic in haplogroup A.

**Table 3:** Analysis of molecular variance within and between Haplogroup A and B

Source of variation	Sum of squares	Variance components	Percentage of variation	P-value
Between haplogroups	546.088	17.66663	76.3	0.001
Within haplogroup	647.745	5.48937	23.7	0.001
Total	1193.833	23.15599		

In an exponentially growing population, the distribution of pairwise differences can provide useful information if the distribution is a Poisson distribution [25]. The gene haplotype network in this scenario resembles a star with all the nodes clustered in time, implying that all coalescent events will take place close to the root and few, if any, will take place later.

### Historical and demographic profile of Sudan desert sheep

**Fig. 2:**
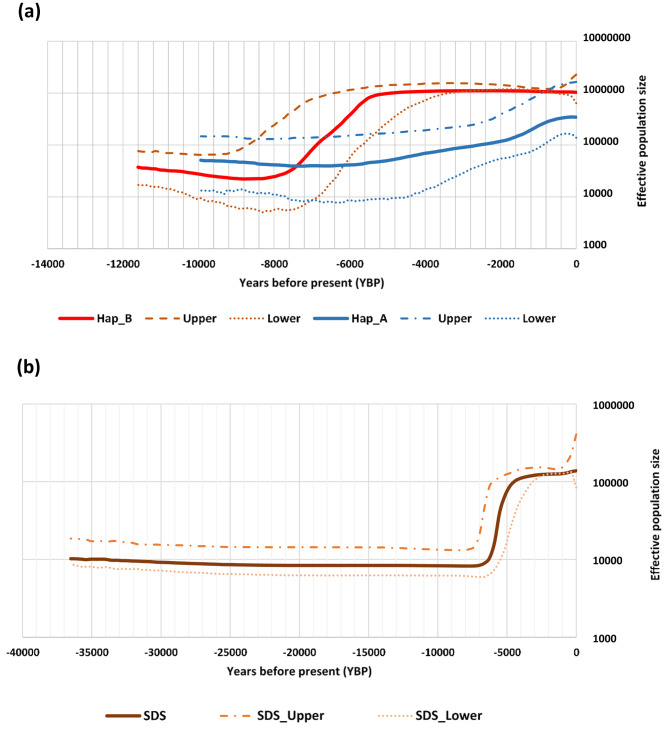
Coalescent Bayesian skyline plots for the a, haplogroup A & B; b All dataset (Sudan desert sheep). Solid lines show median estimate of effective population size. Dotted lines indicate 95% highest posterior density interval (HPD) curves

The mismatch analysis for all haplotypes gave negative values of Tajima’s *D* and Fu’s *Fs* with significant values for all Fu’s *Fs* results. The histograms of mismatch distribution revealed two distinct peaks (bimodal) for all except the haplogroup B (Table 1 and Figure S2). These findings support the recent expansion of Sudan desert sheep breeds. We obtained a better resolution of the demographic history and profile of the study populations by modelling changes in effective population size (*N*_*e*_) through time with the generation of Bayesian Skyline Plots (BSP) for each breed (Hamary, Kabashi and Crossbreed) and the two haplogroups (A and B). As indicated in the materials and methods, we calibrated the BSPs using the cattle mtDNA control region’s molecular rate of evolution (µ). The profiles of the skyline plot for the haplogroups showed that haplogroup B had the highest effective population size. It started to coalesce earlier, at around 11,000 YBP, and started its expansion earlier, at around 8000 YBP, compared to haplogroup A, which started to coalesce at about 10,000 YBP and started to expand at about 6500 YBP (Fig. 2A). Moreover, haplogroup B reached a plateau at around 2000 YBP compared to 150 YBP for haplogroup A. The combined dataset of SDS revealed coalescence, the start of the expansion, and reaching a plateau occurred at around 3700, 700, and 4000 YBP, respectively (Fig. 2B).

**Fig. 3:**
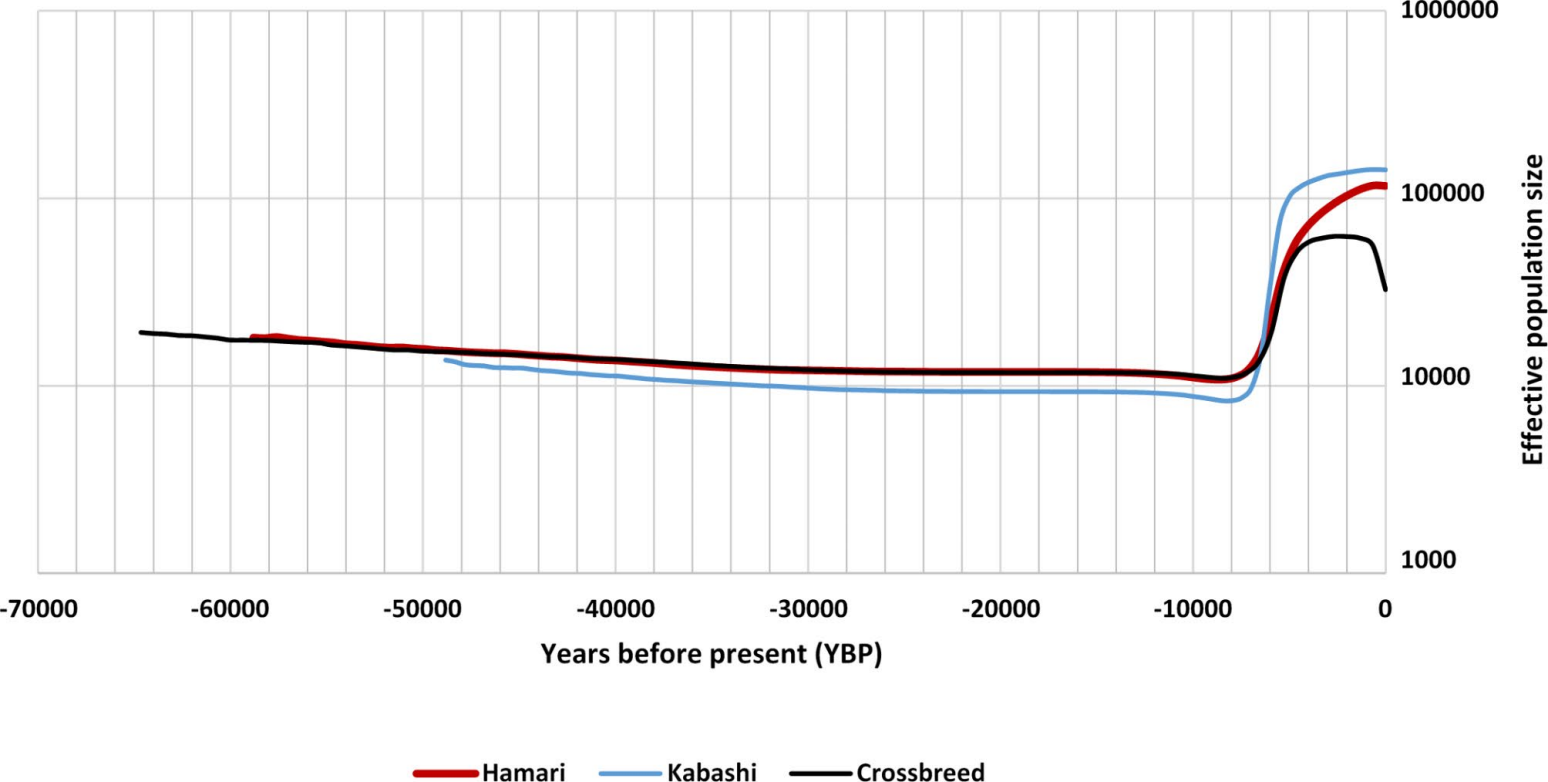
Coalescent Bayesian skyline plots for the Hamary, Kabashi and Crossbreed of Sudan desert sheep. Solid lines show median estimate of effective population size. Dotted lines indicate 95% highest posterior density interval (HPD) curves

On the other hand, the three sheep breed Hamary, Kabashi, and Crossbreed started to expand at around 8000 YBP. Crossbreed was the earliest to coalesce around 6500 YBP, then Hamary at 5900 YBP and Kabashi at 4900 YBP. The *N*_*e*_ of all populations remains constant to the present time, except in Crossbreed population which shows a gradual declining trend from ~ 100 YBP. The highest effective population size was observed in Kabashi, Hamari, and the lowest one in Crossbreed (Fig. 3 and Figure S3).

## Discussion

An analysis of the complete mitochondrial control region sequences of 120 sheep belonging to three Sudan desert sheep (SDG) breeds (Hamary, Kabashi, and Crossbreed) was presented in this study. All SDG are classified as thin-tailed sheep and have been reported to likely share an ancestry with both European and Asian sheep [26].

Our results provide interesting insights about the genetic origin of the crossbred Sudan desert sheep breed, with mtDNA D-loop data supporting predominantly female Hamary origins for the Crossbreed. Indeed, only shared mtDNA D-loop haplotypes were observed between Hamary and Crossbreed, with none observed between the Crossbreed and Kabashi. Thus, though the crossbreeding between Hamary and Kabashi may appear random, it appears to follow a crossbreeding pattern selected by the shepherds.

It is widely acknowledged that domestic sheep have five maternal mitochondrial DNA (mtDNA) lineages (i.e., A, B, C, D, and E), some with distinct geographic distributions. This study revealed widespread occurrences of haplogroup B and, to a smaller extend, of haplgrogroup A in Sudan desert sheep. Similar results were obtained in a previous study that screening 231 Sudan sheep using restriction fragment length polymorphism, where the majority of the sequences belonged to haplogroup B, with only around 10% to haplogroup A [27]. Additionally, a mtDNA control region analysis of 91 domestic sheep from Kenya identified 90 haplogroup B and only one haplogroup A haplotype [28]. A study of 31 Ethiopian domestic sheep identified five (16.12%) haplogroup A and 26 (83.88%) haplogroup B sequences [29]. Interestingly, in Algeria, 87% of Algerian sheep had sequences within haplogroup B, with the remaining belonging to haplogroup C rather than A [30].

The signature of a population expansion in Sudan desert sheep was revealed through a mismatch distributions analysis under spatial expansion assumptions. A negative and significant Fu’s *Fs* value indicated an abundance of rare haplotypes, which is consistent with a recent population expansion or background selection [19]. This finding was further supported by an association between one common haplotype and others with lower frequencies or private haplotypes [17, 25].

Out of all 96 observed haplotypes, 87.5% were unique, indicating significant maternal diversity in the studied populations. Furthermore, most haplotypes were one mutation step away from each other, suggesting recent expansions. The star-like median-joining network, which had several median vectors, indicates the presence of unsampled genotypes or extinct ancestral sequences. This, in association with extensive single haplotypes presence, support little maternal genetic structure within the SDS breeds.

Recent analysis of the control region of mitochondrial DNA (D loop) in 11 indigenous Indian sheep breeds revealed the presence of maternal haplogroups A, B, and C as well as evidence of population expansion [31]. In contrast, in the Mediterranean region and eastern Europe, haplogroups A, B, and C were reported in three sheep breeds from Egypt and two from Italy [32], as well as in two breeds from Hungary [33], with the absence of haplogroups D and E. However, haplogroup D was found in 2.2% of seven Italian sheep breeds, according to [34].

As expected, the NJ phylogenetic tree formed two separate clades representing haplogroup B as the more frequent than haplogroup A. Two major lineages, A and B, and three minor lineages, C, D, and E, have been identified in sheep breeds worldwide [35]. We rationalized the absence of the minor lineages (C, D, and E) by the fact that lineage C is thought to have a limited distribution in semi-desert and steppe regions between 30° and 45° north latitudes However, lineage E is present in Algeria which open the door for further discussion. Additionally, lineage C co-occurs with native fat-tailed breeds, suggesting that the geographic distribution of fat-tailed breeds may be related to the predominance of this lineage [3, 36]. However, lineages D and E in domestic sheep are exceptionally rare and were only reported in the North Caucasus region [4].

Latest evidence on the diversity of the mitochondrial DNA control region, the phylogenetic relationships among African sheep breeds, and their demographic histories reveal that thin tails sheep primarily dominate haplogroup B, which has been further subdivided into B1, B2, and B3, with the Sudan haplogroup belonging to B1. According to [37], the sub-haplogroup B1, primarily from West Africa and Sudan, appears to have had higher dispersal characteristics than other sub-haplogroups. The same study suggests that Sudan may have played a significant role in the dispersion of B1, both southward and westward.

According to [26], the thin-tailed sheep were the first sheep to be introduced into Africa, followed by the fat-tailed sheep through the north-eastern part of the continent and the Horn of Africa. The thin-tailed sheep from the Sudan desert displayed various historical demographic characteristics of interest. Our findings indicate that haplogroup B coalesced before haplogroup A, supporting higher diversity and larger coalescent effective population sizes of haplogroup B compare to haplogroup A. Remarkably, the expansion of Hamary, Kabashi, and their Crossbreed all occurred around the same period. These can be accounted for using the mutation rate of the cattle mtDNA control region, as the mutation rate for sheep is currently unavailable.

## Conclusion

This study has revealed the widespread presence haplogroup B, low mtDNA differentiation of the three Sudan desert sheep, and high maternal diversity among breeds. The results also demonstrate that three breeds, Hamary, Kabashi, and Crossbreed, and the two major haplogroups, A and B, have undergone population expansions in the past, suggesting differences in their demographic histories. The knowledge gained in this study may help improving sheep genetic resource conservation and utilisation. Indeed, they suggest that the Sudan desert sheep may represent a unique genetic resource with two main maternal influence: an ancient one (haplogroup B) and a more recent one (haplogroup A). However, further research is needed to investigate the diversity and linkages between contemporary populations of African sheep and their ancient counterparts to further support the history of Sudan desert sheep proposed here. It is also recommended to identify the genetic and phenotypic characteristics of other local sheep populations from various geographical regions to understand their adaptation to local environmental circumstances.

## Electronic supplementary material

Below is the link to the electronic supplementary material.

**Additional file 1: Table S1:** Analysis of molecular variance within and among breeds of Sudan desert sheep. **Figure S1.** Unrooted NJ tree for Haplogroup A and B of Sudan desert sheep mtDNA D-loop haplotypes. **Figure S2.** Mismatch distribution of pairwise nucleotide differences at the haplogroup level (a, b) and at breed level (c, Hamary, d, Kabashi; e, Crossbreed). **Figure S3.** Coalescent Bayesian skyline plots at the breed level, a, Hamary; b, Kabashi; c, crossbreed.

## Data Availability

The sequences obtained were deposited to the DNA Data Bank of Japan (http://www.ddbj.nig.ac.jp) under accession numbers LC456425 – LC456544.
