# Supplementary material for: Genetic variation and demographic history of Sudan desert sheep reveal two diversified lineages
Source: BMC Genomics. 2023 Mar 16;24:118. doi: 10.1186/s12864-023-09231-6 (PMC10018940; doi:10.1186/s12864-023-09231-6)
Supplement: Supplementary file 1 — Additional file 1: Table S1: Analysis of molecular variance within and among breeds of Sudan desert sheep. Figure S1. Unrooted NJ tree for Haplogroup A and B of Sudan desert sheep mtDNA D-loop haplotypes. Figure S2. Mismatch distribution of pairwise nucleotide differences at the haplogroup level (a, b) and at breed level (c, Hamary, d, Kabashi; e, Crossbreed). Figure S3. Coalescent Bayesian skyline plots at the breed level, a, Hamary; b, Kabashi; c, crossbreed. [file 12864_2023_9231_MOESM1_ESM.docx]

| **Breed** | **Source of variation** | **d.f.** | **Sum of squares** | **Variance components** | **Percentage of variation** | **P-value** |
| --- | --- | --- | --- | --- | --- | --- |
| **Crossbreed** | Among populations | 1 | 18.125 | 0.33305 | 3.26 | 0.32747 |
|  | Among individuals within populations | 1 | 4.737 | -0.14261 | -1.40 | 0.91300 |
|  | Within individuals | 117 | 1173.471 | 10.02967 | 98.14 |  |
|  | **Total** | **119** | **1196.333** | **10.22011** |  |  |
| **Kabashi** | Among populations | 1 | 6.481 | 6.481 | -2.39 | 1.00000 |
|  | Among individual within populations | 1 | 16.382 | 0.18220 | 1.83 | 0.10655 |
|  | Within individuals | 117 | 1173.471 | 10.02967 | 100.56 | 0.27859 |
|  | **Total** | **119** | **1196.333** | **1196.333** | **9.97344** |  |
| **Hamary** | Among populations | 1 | 7.701 | -0.20115 | -2.00 | 0.65689 |
|  | Among individuals within populations | 1 | 15.161 | 0.21418 | 2.13 | 0.13001 |
|  | Within individuals | 117 | 1173.471 | 10.02967 | 99.87 | 0.28641 |
|  | **Total** | **119** | **1196.333** | **10.04270** |  |  |

**Table S1: Analysis of molecular variance within and among breeds of Sudan desert sheep**

**Clade B**

**Clade A**

**Figure S1** Unrooted NJ tree for Haplogroup A and B of Sudan desert sheep mtDNA D-loop haplotypes.

**a,**

**b,**

**c,**

**d,**

**c,**

**e,**

**Figure S2** Mismatch distribution of pairwise nucleotide differences at the haplogroup level (a, b) and at breed level (c, Hamary, d, Kabashi; e, Crossbreed).


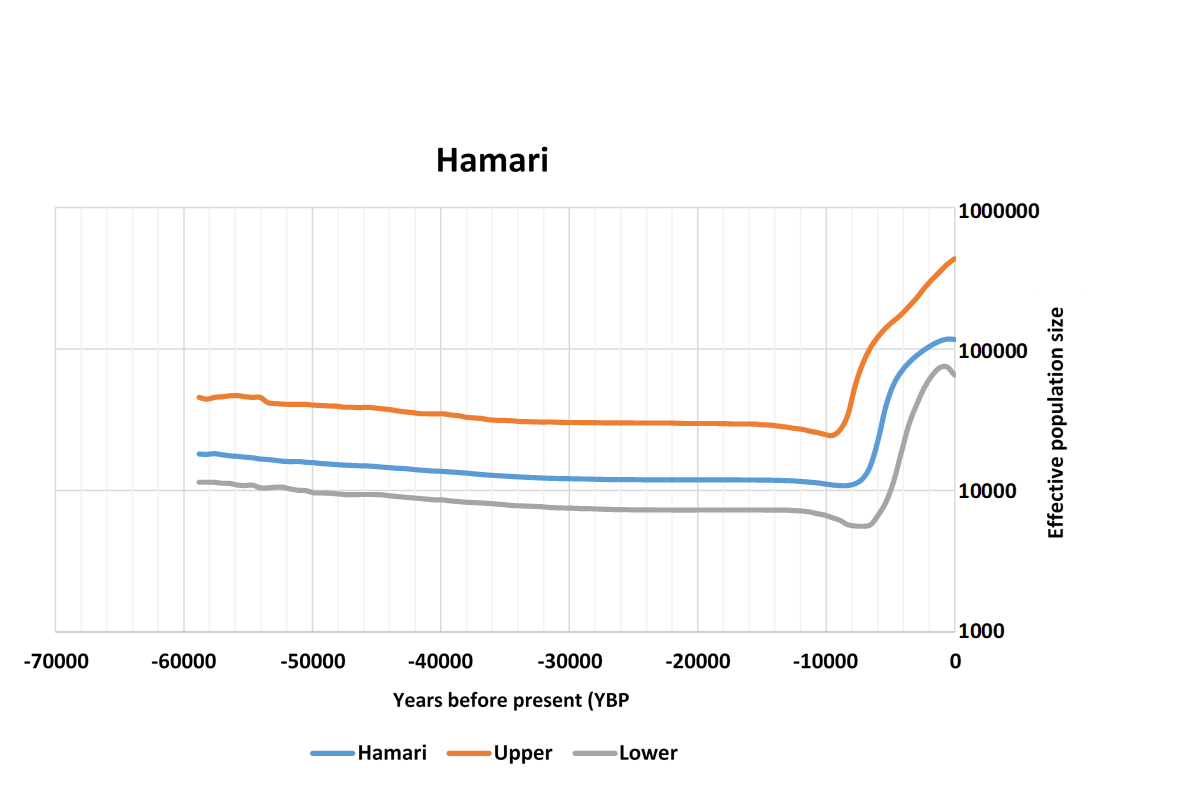
**a,**

**b,**

**c,**

**Figure S3** Coalescent Bayesian skyline plots at the breed level, a, Hamary; b, Kabashi; c, crossbreed. Solid lines show median estimate of effective population size. Dotted lines indicate 95% highest posterior density interval (HPD) curves.
